# A Low-SNR DOA Estimation Model Based on Sequential and Convolutional Feature Fusion

**DOI:** 10.3390/s26103093

**Published:** 2026-05-13

**Authors:** Wenchao He, Yiran Shi, Jianchao Wang, Hongxi Zhao

**Affiliations:** 1 School of Mechanical and Electrical Engineering, Changchun Humanities and Sciences College, Changchun 130118, China; hewc23@mails.jlu.edu.cn; 2 College of Communication Engineering, Jilin University, Changchun 130012, China; w1748281912@163.com (J.W.); hxzhao24@mails.jlu.edu.cn (H.Z.)

**Keywords:** array signal processing, direction-of-arrival estimation, deep learning, ResNet, Mamba, feature fusion, uniform linear array

## Abstract

This paper proposes a novel hybrid deep learning framework for direction-of-arrival (DOA) estimation using a uniform linear array. Direction of Arrival estimation is a fundamental problem in array signal processing with critical applications in radar, sonar, wireless communications, and speech processing. Traditional methods like MUSIC and ESPRIT provide high resolution but suffer from high computational complexity and poor performance in low signal-to-noise ratio (SNR) environments. Recent advances in deep learning have shown promise in improving DOA estimation accuracy and robustness. The framework synergistically combines a ResNet-based feature extractor with a Mamba state-space model through a feature fusion mechanism. The ResNet branch extracts high-level spatial features from the covariance matrix, while the Mamba branch captures long-range dependencies and sequential patterns. These complementary features are fused and then passed to an MLP for DOA regression. Extensive experiments on simulated datasets demonstrate that, at low SNRs, our fusion model significantly outperforms traditional methods such as MUSIC and ESPRIT, as well as other baseline models, in terms of both estimation accuracy and computational efficiency. Quantitatively, at SNR = −5 dB, the proposed method reduces the RMSE by 41.6% compared to MUSIC.

## 1. Introduction

Direction-of-arrival (DOA) estimation is a fundamental problem in array signal processing, with critical applications in radar, sonar, wireless communications, and speech processing. Accurately determining the directions of signal sources is essential for target localization, beamforming, and interference suppression in both civilian and military applications. With the evolution of wireless communication systems towards higher frequencies and larger-scale antenna arrays, the demand for accurate and computationally efficient DOA estimation algorithms has become increasingly urgent [1].

Traditional high-resolution DOA estimation algorithms, such as MUSIC [2] and ESPRIT [3], are based on the subspace decomposition of the array covariance matrix. These methods achieve excellent angular resolution when the sample covariance matrix is accurately estimated and the signal and noise subspaces are well separated. However, their performance degrades considerably in adverse scenarios, such as low-SNR environments, short observation intervals, and coherent multipath propagation [4]. Specifically, MUSIC requires both an accurate estimate of the number of sources and a reliable covariance matrix, and thus becomes vulnerable when noise contamination obscures the subspace structure [5]. ESPRIT eliminates spectral searching, but its dependence on rotational invariance makes it sensitive to array calibration errors and mutual coupling effects [6]. Although a number of improved methods have been developed, including covariance reconstruction and denoising strategies prior to subspace decomposition [7], they still inherit the limitations of the subspace-based framework and often increase computational burden.

Most of the above discussion concerns conventional passive ULA-based DOA estimation, which is also the main focus of this work. In a passive ULA system, the received signals are collected directly by a physical linear array, and the associated steering vector is determined solely by the geometry of the receive array. By contrast, MIMO radar employs both transmit and receive arrays, and after waveform separation, the received data can be interpreted through a virtual array model formed jointly by the transmit and receive apertures, typically described using a Kronecker-product-based steering formulation [8]. As a result, ULA-based DOA estimation and MIMO radar DOA estimation differ not only in array representation, but also in signal modeling and data structure. Specifically, the former is established directly on the spatial samples of a physical receive array, whereas the latter is formulated on the basis of a virtual array generated from the transmit-receive configuration. This distinction has led to different model designs and signal processing strategies in the DOA literature. Nevertheless, despite these differences in array formulation, both ULA and MIMO DOA estimation ultimately aim to infer angular information from structured array observations, which has motivated the development of data-driven methods that can learn effective representations directly from measurement data.

In recent years, deep learning has emerged as a powerful alternative paradigm for DOA estimation, offering improved robustness and significantly reduced computational complexity after an offline training phase [9,10,11]. Liu et al. proposed a complex-valued convolutional network-based DOA estimation method that vectorizes the upper triangular elements of the covariance matrix as input to the network, enabling direct regression of multiple source angles [12]. This approach avoids complex feature engineering and offers low computational latency when the network scale is appropriate. However, this input processing method flattens structured data into a global vector, disrupting the inherent spatial proximity relationships between array elements within the covariance matrix. Furthermore, as the number of array elements increases, the number of network parameters grows quadratically, making the model prone to overfitting and difficult to scale to large arrays [13].

Convolutional neural networks have been increasingly applied to DOA estimation due to their ability to preserve spatial structure [14,15,16,17,18]. Addressing DOA estimation in dynamic scenarios, Burghal et al. proposed a sequential modeling method based on recurrent neural networks (RNNs), utilizing RNNs to model the temporal dependencies between snapshots for tracking moving targets [19]. This approach effectively leverages temporal information across multiple snapshots, demonstrating better tracking performance compared to traditional methods in moving target scenarios. However, RNNs suffer from vanishing gradient problems when processing long sequences, making it difficult to capture dependencies between distant snapshots [20]. Moreover, this method simply concatenates the array data from each snapshot as input, failing to fully utilize the spatial structure information between elements within the same snapshot, which limits its estimation accuracy under low SNR conditions. The recently introduced Mamba architecture offers linear-time sequence modeling with powerful long-range dependency capture capabilities, providing a promising alternative for sequential processing [21,22,23].

To better position our work within the existing literature, Table 1 summarizes representative deep learning-based DOA estimation methods along with their advantages and disadvantages.

To address the limitations of traditional subspace methods, which suffer severe performance degradation under low SNR and limited snapshot conditions, the drawbacks of fully-connected network-based methods that disrupt spatial proximity and incur excessive parameters, and the shortcomings of RNN-based methods that suffer from vanishing gradients and neglect spatial structure information, this paper proposes a hybrid deep learning framework integrating ResNet, Mamba, and MLP for DOA estimation with uniform linear arrays. This framework processes the covariance matrix as structured 2D input through a ResNet branch, leveraging its local connectivity to preserve spatial proximity between elements and control the parameter count, thereby addressing the flaws of fully-connected approaches. It performs sequential modeling along the array dimension through a Mamba branch, utilizing a selective state space mechanism to capture long-range dependencies and phase progression patterns, avoiding the vanishing gradient problem of RNNs and compensating for the neglect of spatial structure information. Finally, an MLP layer performs nonlinear fusion and regression on the features extracted by both branches, achieving complementary enhancement of local spatial features and global sequential features. This enables the model to simultaneously utilize both types of information in low SNR scenarios, where signal characteristics are severely corrupted by noise, resulting in more robust DOA estimation performance. The main contributions of this paper are summarized as follows:

- **A Novel ResNet-Mamba Hybrid Architecture for DOA Estimation:** We propose a hybrid deep learning framework that synergistically integrates a ResNet branch for spatial feature extraction and a Mamba branch for sequential feature modeling. The ResNet branch processes the covariance matrix as a 2D image to capture local spatial correlations between array elements, while the Mamba branch treats the array data as a sequence along the sensor dimension to model long-range dependencies and phase progression patterns. This dual-branch design enables comprehensive feature extraction that neither architecture could achieve independently, providing a principled solution for DOA estimation that leverages both spatial and sequential inductive biases.
- **Feature Fusion with Layer Normalization Optimization:** To effectively combine the complementary features from both branches, we design a feature fusion mechanism incorporating layer normalization before concatenation. This normalization step stabilizes training by ensuring that features from different branches have comparable scales and distributions, preventing one branch from dominating the gradient flow. The fused 1024-dimensional representation preserves both local spatial correlations (from ResNet) and global sequential patterns (from Mamba), enabling the subsequent MLP regressor to learn optimal combinations for accurate DOA prediction. This Wdesign provides an effective solution for multi-branch feature fusion in DOA estimation.
- **Comprehensive Experimental Validation and Benchmarking:** We conduct extensive experiments on simulated datasets with a 10-element uniform linear array across a wide range of SNR conditions (−5 dB to 10 dB) and snapshot settings. The proposed fusion model is systematically compared against traditional methods (MUSIC, ESPRIT) and deep learning baselines. Results demonstrate that our model achieves superior accuracy, particularly in the low SNR regime, with significant RMSE improvements compared to existing approaches. We further validate the model’s robustness through ablation studies and snapshot efficiency evaluation, providing comprehensive empirical evidence for the effectiveness of the proposed approach.

## 2. Methods

### 2.1. Signal Model Construction

In this experiment, we consider *M* far-field narrowband, uncorrelated signal sources denoted by

(1) $$\theta ={\left[\theta_{1},\theta_{2},\ldots ,\theta_{M}\right]}^{⊤}$$

We assume a uniform linear array with N=10 sensors, where the sensors are equally spaced at half-wavelength intervals. The sensor positions are indexed with respect to the first sensor:

(2) $$L=\left\{0,d,2d,\ldots ,9d\right\},\;d=\frac{\lambda }{2}$$

For the *n*-th sensor (n=1,2,…,N), the received signal at time *t* is

(3) $$\psi_{n}\left(t\right)=\sum_{k=1}^{M}a_{n}\left(\theta_{k}\right)s_{k}\left(t\right)+\mu_{n}\left(t\right)$$

where

(4) $$a_{n}\left(\theta_{k}\right)=e^{-j\frac{2\pi }{\lambda }\left(n-1\right)d\sin\left(\theta_{k}\right)}$$

Based on these equations, we can define the observation vector as

(5) $$\psi \left(t\right)={\left[\psi_{1}\left(t\right),\psi_{2}\left(t\right),\ldots ,\psi_{N}\left(t\right)\right]}^{T}=A\left(\theta \right)s\left(t\right)+\mu \left(t\right)$$

where

(6) $$\begin{array}{cc} A(\theta ) & =\left[a\left(\theta_{1}\right),a\left(\theta_{2}\right),\ldots ,a\left(\theta_{M}\right)\right]\in C^{N\times M}, \end{array}$$

(7) $$\begin{array}{cc} a(\theta_{k}) & ={\left[a_{1}\left(\theta_{k}\right),a_{2}\left(\theta_{k}\right),\ldots ,a_{N}\left(\theta_{k}\right)\right]}^{T}\in C^{N\times 1}, \end{array}$$

(8) $$\begin{array}{cc} s(t) & ={\left[s_{1}\left(t\right),s_{2}\left(t\right),\ldots ,s_{M}\left(t\right)\right]}^{T}\in C^{M\times 1}, \end{array}$$

(9) $$\begin{array}{cc} \mu (t) & ={\left[\mu_{1}\left(t\right),\mu_{2}\left(t\right),\ldots ,\mu_{N}\left(t\right)\right]}^{⊤}\in C^{N\times 1} \end{array}$$

For a uniform linear array with half-wavelength spacing d=λ/2, the steering vector $a(\theta_{k})$ takes the canonical Vandermonde form:

(10) $$a\left(\theta_{k}\right)=\left[\begin{array}{c} 1\\ e^{-j\pi \sin(\theta_{k})}\\ e^{-j2\pi \sin(\theta_{k})}\\ \vdots \\ e^{-j9\pi \sin(\theta_{k})} \end{array}\right]\in C^{N\times 1}$$

#### Covariance Matrix Calculation

The covariance matrix of the received signals is computed from *L* snapshots as

(11) $${\hat{R}}_{\psi \psi }=\frac{1}{L}\sum_{t=1}^{L}\psi \left(t\right)\psi^{H}\left(t\right)\in C^{N\times N}$$

For uncorrelated sources, the theoretical covariance matrix has the structure

(12) $$R_{\psi \psi }=E\left[\psi \left(t\right)\psi^{H}\left(t\right)\right]=AR_{ss}A^{H}+\xi_{n}^{2}I\in C^{10\times 10}$$

Based on the above equations, we can effectively suppress the white noise by computing the expectation of the signal and noise covariance $E[\psi \left(t\right)\psi^{H}\left(t\right)]$.

Since complex-valued data cannot be directly fed into neural networks for effective feature extraction, we separate the real and imaginary parts of the covariance matrix and stack them along a new dimension, forming a 3D tensor:

(13) Rinput∈R10×10×2,whereRinput(:,:,0)=Re(R^ψψ),Rinput(:,:,1)=Im(R^ψψ)

In this 3D tensor, the first dimension (index *i*) corresponds to the array element index, the second dimension (index *j*) corresponds to the array element index, and the third dimension (index c∈{0,1}) represents the channel, with channel 0 containing the real parts and channel 1 containing the imaginary parts. Thus, the elements $R_{\mathrm{input}}\left(i,j,0\right)$ and $R_{\mathrm{input}}\left(i,j,1\right)$ together constitute the complex covariance ${\hat{R}}_{\psi \psi }\left(i,j\right)=R_{\mathrm{input}}\left(i,j,0\right)+jR_{\mathrm{input}}\left(i,j,1\right)$.

For sequence modeling with the Mamba network, we reshape this 3D tensor into a 2D representation where each row corresponds to an array element and contains the concatenated real and imaginary parts of the covariance matrix for that array element:

(14) $$\begin{array}{cc} X_{seq}\in R^{10\times 20},\;\mathrm{where}\;X_{seq}\left(i,:\right)= & [R_{\mathrm{input}}\left(i,1,0\right),\ldots ,R_{\mathrm{input}}\left(i,10,0\right),\\ \; & \;\;R_{\mathrm{input}}\left(i,1,1\right),\ldots ,R_{\mathrm{input}}\left(i,10,1\right)] \end{array}$$

### 2.2. Construction of the Deep Learning Model

#### 2.2.1. ResNet Branch: Spatial Feature Extraction

The ResNet branch is designed to extract high-level spatial features from the 10×10×2 covariance matrix representation. The input to this branch is the real-valued tensor $R_{\mathrm{input}}\in R^{10\times 10\times 2}$, where the first two dimensions (10 × 10) represent the correlation between all pairs of array elements, and the third dimension contains the real and imaginary parts as separate channels.

In ResNet, data is represented as a multi-dimensional tensor where convolutional kernels slide over spatial dimensions to extract local patterns. The hierarchical relationships between features are learned through successive convolutional layers. Unlike plain convolutional networks, ResNet introduces skip connections that allow gradients to flow directly through the network, mitigating the degradation problem and enabling the training of deeper architectures.

For the covariance matrix $R_{\mathrm{input}}$, each element $R_{\mathrm{input}}\left(i,j,c\right)$ represents the correlation between the *i*-th and *j*-th array elements for channel *c* (with c=0 for real part and c=1 for imaginary part). The indices *i* and *j* both range from 1 to 10, corresponding to the 10 array elements.

The feature extraction process begins with a data adapter that interpolates the 10×10 input to 224×224 using bilinear interpolation, producing $H^{\left(0\right)}\in R^{224\times 224\times 2}$.

The first layer consists of a convolution with batch normalization and ReLU activation, followed by max pooling:

(15) $$H^{\left(1\right)}=\mathrm{MaxPool}\left(\mathrm{ReLU}\left(\mathrm{BN}\left(W^{\left(1\right)}*H^{\left(0\right)}\right)\right)\right)$$

where $W^{\left(1\right)}\in R^{7\times 7\times 2\times 48}$ is the convolutional kernel, and the max pooling uses a 3×3 kernel with stride 2 and padding 1. This reduces the spatial dimensions from 224×224 to 56×56.

The core building block of the ResNet branch is the bottleneck residual block. For an input x, the bottleneck block computes a residual mapping F(x) and adds it to the input via a skip connection:

(16) y=ReLU(x+F(x))

when the input and output dimensions differ, a projection skip connection with a 1×1 convolution is used:

(17) y=ReLU(Projection(x)+F(x))

the residual mapping F(x) consists of three convolutional layers:

(18) $$F\left(x\right)=\mathrm{BN}\left(W_{3}\mathrm{ReLU}\left(\mathrm{BN}\left(W_{2}\mathrm{ReLU}\left(\mathrm{BN}\left(W_{1}x\right)\right)\right)\right)\right)$$

with kernel dimensions:

(19) $$\begin{array}{c} W_{1}\in R^{1\times 1\times C_{\mathrm{in}}\times C_{\mathrm{mid}}}, \end{array}$$

(20) $$\begin{array}{c} W_{2}\in R^{3\times 3\times C_{\mathrm{mid}}\times C_{\mathrm{mid}}}, \end{array}$$

(21) $$\begin{array}{c} W_{3}\in R^{1\times 1\times C_{\mathrm{mid}}\times \left(4\cdot C_{\mathrm{mid}}\right)}. \end{array}$$

The ResNet branch consists of 3 stages, comprising 3, 4, and 6 bottleneck residual blocks, respectively. The bottleneck parameters $\left(C_{\mathrm{in}},C_{\mathrm{mid}}\right)$ for each stage are (48,48), (192,96), and (384,192), producing output channels of 192, 384, and 768.

The spatial dimensions are transformed as follows:

(22) $$\begin{array}{cc} 224\times 224 & \overset{\mathrm{Conv}1\;+\;\mathrm{pool}}{\to }56\times 56\overset{\mathrm{Stage}1}{\to }28\times 28\\ \; & \overset{\mathrm{Stage}2}{\to }14\times 14\overset{\mathrm{Stage}3}{\to }7\times 7 \end{array}$$

The channel dimensions evolve as:

(23) $$\begin{array}{cc} 2 & \overset{\mathrm{Conv}1}{\to }48\overset{\mathrm{Stage}1}{\to }192\overset{\mathrm{Stage}2}{\to }384\overset{\mathrm{Stage}3}{\to }768 \end{array}$$

After the third stage, global average pooling is performed to integrate the spatial features into a global feature vector:

(24) $$f_{\mathrm{res}}=\frac{1}{7\times 7}\sum_{i=1}^{7}\sum_{j=1}^{7}H_{i,j}^{\left(3\right)}\in R^{768}$$

where $H^{\left(3\right)}$ is the output of Stage 3. This 768-dimensional vector serves as the spatial feature representation for subsequent fusion with temporal features. The skip connections in each bottleneck block enable effective gradient propagation, preventing gradient vanishing and enabling stable training.

#### 2.2.2. Mamba Branch: Sequential Feature Extraction

The Mamba branch adopts a dual-branch structure, consisting of a core path for sequential feature extraction and a gate path for adaptive information filtering. The branch is designed to capture sequential dependencies and long-range patterns along the array dimension. Unlike CNNs that operate on local neighborhoods through convolutional kernels, Mamba’s selective state-space mechanism can model interactions across the entire sequence, making it particularly suitable for capturing the global structure of array covariance matrices.

In this branch, data is represented as a sequence $X_{seq}\in R^{10\times 20}$, where each position in the sequence corresponds to an array element and contains a 20-dimensional feature vector. This representation preserves the natural ordering of elements along the linear array.

The feature vector for each position *i* is defined as the *i*-th row of the reshaped covariance matrix:

(25) $$\begin{array}{cc} h_{i}=X_{seq}\left[i,:\right]= & [R_{\mathrm{input}}\left(i,1,0\right),\ldots ,R_{\mathrm{input}}\left(i,10,0\right),\\ \; & \;\;R_{\mathrm{input}}\left(i,1,1\right),\ldots ,R_{\mathrm{input}}\left(i,10,1\right)]\in R^{20} \end{array}$$

By treating each row of the reshaped covariance matrix as the input feature, the Mamba network is able to capture the sequential relationships between different array elements.

The practical implementation in our model begins with an input projection layer that maps the 20-dimensional input features to a higher-dimensional space:

(26) $$H^{\left(0\right)}=X_{seq}W_{proj}+b_{proj}\in R^{10\times 128},\;W_{proj}\in R^{20\times 128}$$

The core of the branch consists of four stacked Mamba blocks, each designed to process sequential information through a combination of convolutions and gating mechanisms. For an input sequence $H\in R^{L\times D}$ (where L=10 and D=128), each Mamba block operates as follows:

First, layer normalization is applied to stabilize training:

(27) $$H_{ln}=\mathrm{LayerNorm}\left(H\right)$$

The normalized input is then projected to an expanded dimension with a gating mechanism:

(28) $$H_{proj}=H_{ln}W_{proj2}+b_{proj2},\;W_{proj2}\in R^{128\times 256}$$

The projected features are split into a core path and a gate path:

(29) $$H_{core},H_{gate}=\mathrm{split}\left(H_{proj}\right)\;\mathrm{each}\;\in R^{L\times 128}$$

The core path then undergoes a 1D depthwise convolution to incorporate local context:

(30) $$H_{conv}={\mathrm{DepthwiseConv}1D}_{k=3}{\left(H_{core}^{⊤}\right)}^{⊤}$$

After convolution, the GELU (Gaussian Error Linear Unit) activation function is applied:

(31) $$H_{act}=\mathrm{GELU}\left(H_{conv}\right)$$

Simultaneously, the gate path passes through a sigmoid activation to produce gating values between 0 and 1:

(32) $$H_{gate}=\sigma \left(H_{gate}\right)$$

The gating mechanism then controls information flow by element-wise multiplication:

(33) $$H_{gated}=H_{gate}⊙H_{act}$$

The gated features are then projected back to the original dimension:

(34) $$H_{out}=\mathrm{Dropout}\left(H_{gated}W_{out}+b_{out}\right),\;W_{out}\in R^{256\times 128}$$

Finally, a residual connection adds the original input to the processed output:

(35) $$Y=H+H_{out}$$

The four Mamba blocks are stacked sequentially, with each block taking the output of the previous block as input.

After processing through four Mamba blocks, we apply global pooling across the sequence dimension to obtain a fixed-size representation that captures the overall sequential patterns:

(36) $$\begin{array}{cc} h_{mean} & =\frac{1}{L}\sum_{i=1}^{L}h_{i}\in R^{128}, \end{array}$$

(37) $$\begin{array}{cc} h_{max} & =\max_{i=1}^{L}h_{i}\in R^{128}, \end{array}$$

(38) $$\begin{array}{cc} f_{mam} & =\mathrm{Concat}\left(h_{mean},h_{max}\right)\in R^{256} \end{array}$$

#### 2.2.3. Feature Fusion Module

To combine the complementary spatial features from the ResNet branch ($f_{res}\in R^{768}$) and the sequential features from the Mamba branch ($f_{mam}\in R^{256}$), we employ a simple feature fusion mechanism. Both feature vectors are first normalized using layer normalization to stabilize training:

(39) $$\begin{array}{cc} f_{res\_norm} & ={\mathrm{LayerNorm}}_{768}\left(f_{res}\right), \end{array}$$

(40) $$\begin{array}{cc} f_{mam\_norm} & ={\mathrm{LayerNorm}}_{256}\left(f_{mam}\right) \end{array}$$

The normalized features are then fused by direct concatenation along the feature dimension:

(41) $$f_{fus}=\mathrm{Concat}\left(f_{res\_norm},f_{mam\_norm}\right)\in R^{1024}$$

#### 2.2.4. MLP Layer and Backpropagation

Next, we input $f_{fus}$ into an MLP model for training and updating the weights of the network. The MLP consists of an input layer, hidden layers, and an output layer.

The forward propagation process of the MLP is as follows:

(42) $$h^{\left(0\right)}=f_{fus}$$

For each hidden layer l=1,2, we compute:

(43) $$\begin{array}{c} z^{\left(l\right)}=W^{\left(l\right)}h^{\left(l-1\right)}+b^{\left(l\right)}, \end{array}$$

(44) $$\begin{array}{c} h^{\left(l\right)}=\mathrm{GELU}(\mathrm{LayerNorm}\left(z^{\left(l\right)}\right)), \end{array}$$

(45) $$\begin{array}{c} h^{\left(l\right)}=\mathrm{Dropout}(h^{\left(l\right)}) \end{array}$$

with weight dimensions:

(46) $$\begin{array}{c} W^{\left(1\right)}\in R^{1024\times 512}, \end{array}$$

(47) $$\begin{array}{c} W^{\left(2\right)}\in R^{512\times 256} \end{array}$$

When l=3, this layer is the output layer, and $z^{\left(3\right)}$ is the output of this layer:

(48) $$z^{\left(3\right)}=W^{\left(3\right)}h^{\left(2\right)}+b^{\left(3\right)},\;W^{\left(3\right)}\in R^{256\times 2}$$

The final output $\hat{y}$ contains the estimated DOA angles for the two sources:

(49) $$\hat{y}=z^{\left(3\right)}\in R^{M},\;M=2$$

The model is trained using the Root Mean Squared Error (RMSE) loss function, which is defined as:

(50) $$L=\sqrt{\frac{1}{B\cdot M}\sum_{b=1}^{B}\sum_{i=1}^{M}{\left({\hat{y}}_{i}^{\left(b\right)}-y_{i}^{\left(b\right)}\right)}^{2}}$$

where *B* is the batch size, *M* is the number of sources (ranging from 1 to 3 depending on the scenario), ${\hat{y}}_{i}^{\left(b\right)}$ is the predicted DOA angle for the *i*-th source in the *b*-th sample, and $y_{i}^{\left(b\right)}$ is the corresponding ground truth angle. The RMSE loss provides a balanced measure of estimation error that penalizes larger deviations more severely.

## 3. Experiments and Results

**Figure 1:**
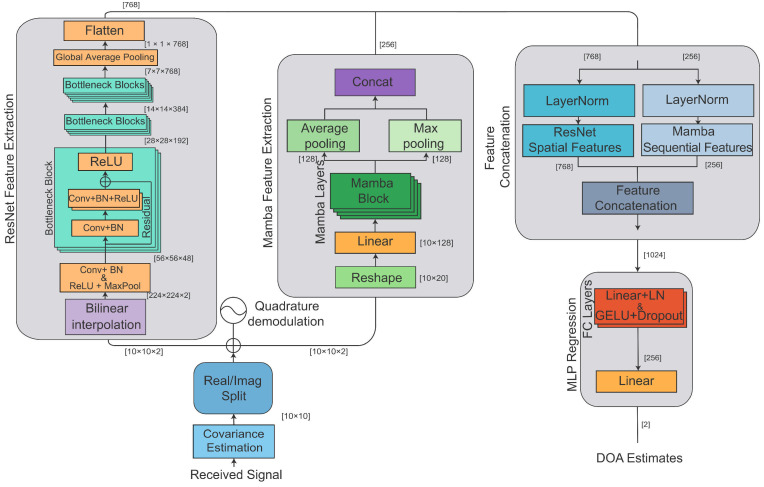
Flowchart of the proposed method.

The overall architecture of the proposed hybrid deep learning framework is illustrated in Figure 1.

Detailed illustration of Figure 1: The proposed framework consists of the following main modules: 1. Input Data and Preprocessing: The received signals from the uniform linear array (ULA) first undergo covariance matrix estimation, producing a complex covariance matrix of size [10×10]. The real and imaginary parts are then separated and stacked to form a 3D tensor of size [10×10×2]. For the ResNet branch, this tensor is bilinearly interpolated to a fixed size of 224×224×2. For the Mamba branch, the tensor is reshaped into a 2D sequential format of size [10×20]. 2. ResNet Feature Extraction (Spatial Features): The ResNet module extracts spatial features from the interpolated covariance matrix. The first convolutional layer with batch normalization and ReLU activation, followed by max pooling, transforms the input to 56×56×48. It then passes through three stages of bottleneck residual blocks, with feature map dimensions progressing as: 56×56×48→28×28×192→14×14×384→7×7×768. A global average pooling layer then reduces the spatial features to a 768-dimensional vector. 3. Mamba Block (Sequential Features): The Mamba block processes sequential features through multiple Mamba layers. The input sequence of size [10×20] is first projected to [10×128] via a linear layer. After passing through four stacked Mamba blocks, global mean pooling and max pooling are applied across the sequence dimension, and their results are concatenated to produce a 256-dimensional feature vector. 4. Feature Concatenation and Fusion: The outputs from the ResNet module (768 dimensions) and the Mamba module (256 dimensions) are each normalized by layer normalization, then concatenated to form a 1024-dimensional fused feature vector. 5. MLP Regression Head: The fused features are passed through an MLP regression head consisting of two hidden layers (1024 → 512 → 256) with LayerNorm, GELU activation, and Dropout for regularization. The final linear layer (256 → 2) outputs the DOA estimates. 6. Output: The network outputs the estimated DOA angles directly as regression values without spectral peak searching.

### 3.1. Implementation Details for Reproducibility

To ensure the reproducibility of our proposed method, we provide detailed specifications of the training dataset composition and model parameter counts.

#### 3.1.1. Training Dataset Composition

The dataset consists of 60,000 simulated samples generated using a 10-element uniform linear array with half-wavelength spacing. The key parameters are as follows:

- Number of sources: Variable
- SNR range: −5 dB to 10 dB (values include −5, 0, 5, and 10 dB)
- Number of snapshots: 256 per sample
- DOA angles: Randomly sampled from the range [−60°, 60°]
- Training/validation split: 90% for training and 10% for validation
- Batch size: 32
- Number of epochs: 100
- Learning rate: 0.0001 with cosine annealing scheduler
- Optimizer: AdamW with weight decay of $1\times 10^{-4}$

#### 3.1.2. Model Parameter Counts

We conducted experiments on a large-scale dataset containing 60,000 samples. The input data were generated using a ten-element uniform linear array with half-wavelength spacing. The detailed model configurations and trainable parameter counts for each module of the proposed fusion model are summarized in Table 2, Table 3, Table 4, and Table 5, respectively.

### 3.2. Results for Single Source Scenario

**Figure 2:**
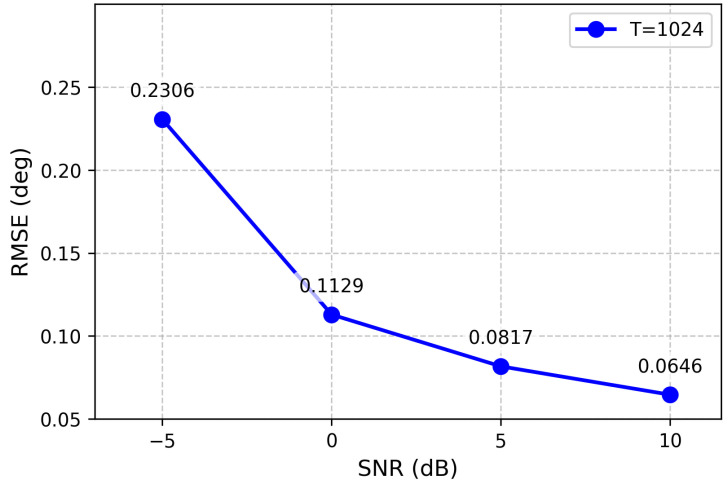
RMSE of the proposed method under different SNR levels (single source).

To evaluate the performance of the proposed model in single-source scenarios, we conducted comprehensive experiments across four different SNR levels (−5, 0, 5, and 10 dB) and computed the corresponding RMSE of the estimated DOAs. The results are presented in Figure 2.

**Figure 3:**
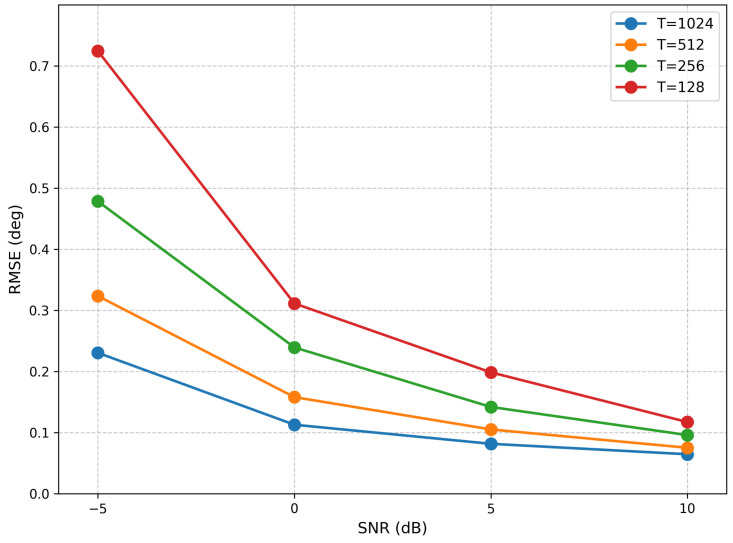
RMSE from Different Snapshots (single source).

Furthermore, to evaluate the performance under different snapshot conditions, we conducted experiments with four distinct snapshot settings (1024, 512, 256, and 128) and computed the corresponding RMSE. The results, presented in Figure 3, clearly demonstrate the impact of snapshot number on estimation accuracy.

### 3.3. Results for Two-Source Scenario

**Figure 4:**
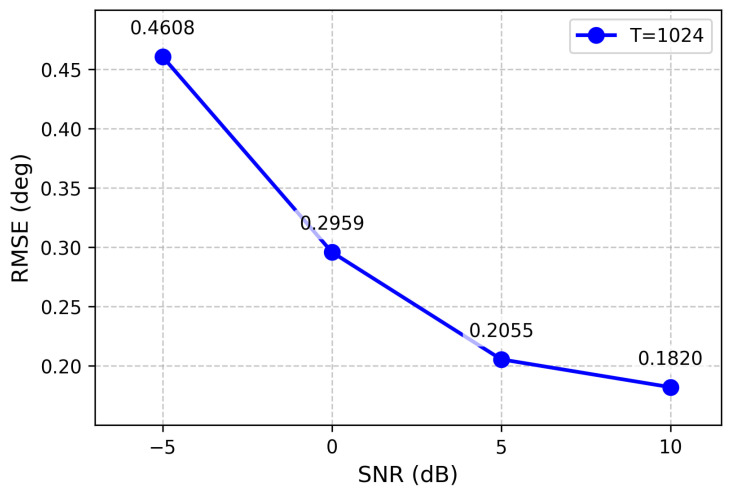
RMSE of the proposed method under different SNR levels (two sources).

The performance of the proposed model in two-source scenarios was evaluated through comprehensive experiments across four different SNR levels (−5, 0, 5, and 10 dB), with the corresponding RMSE of the estimated DOAs computed accordingly. Figure 4 summarizes these results, clearly demonstrating the relationship between SNR and estimation accuracy.

**Figure 5:**
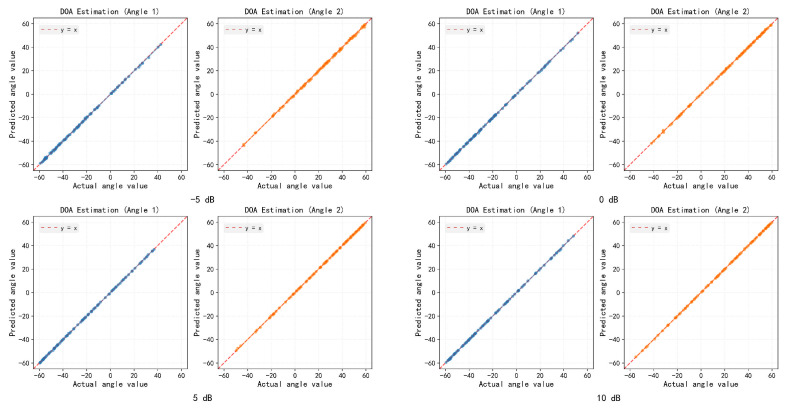
Scatter plots of the proposed method under different SNR levels (two sources).

To visually examine the estimation behavior, we plot the scatter distribution of the estimated DOAs for 400 two-source samples at −5, 0, 5, and 10 dB, as depicted in Figure 5.

**Figure 6:**
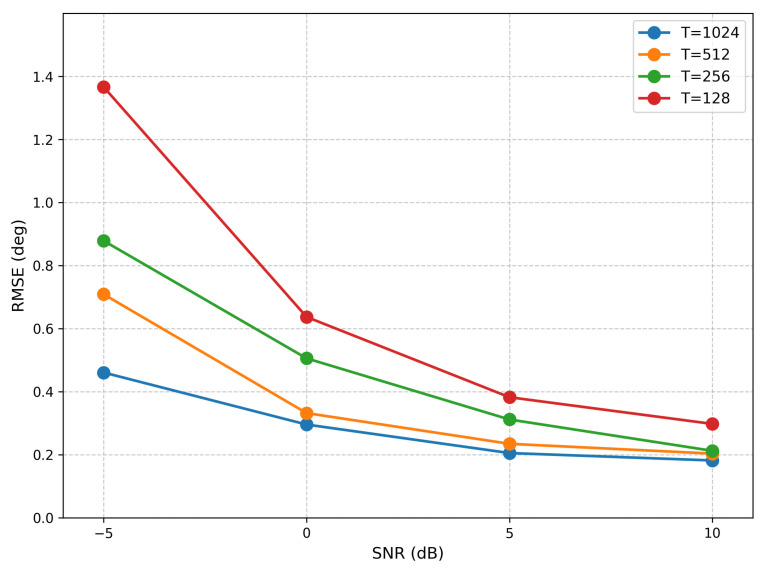
RMSE from Different Snapshots (two sources).

The impact of snapshot number on estimation accuracy is examined in Figure 6, which reports the RMSE under four distinct snapshot settings (1024, 512, 256, and 128). The results confirm that the proposed method maintains robust performance even with limited snapshots.

**Figure 7:**
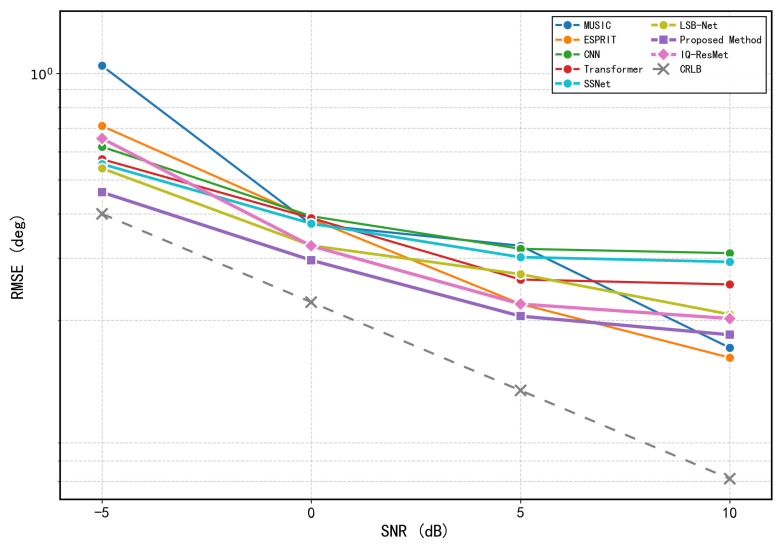
RMSE comparison of different methods under various SNR conditions (two sources).

**Figure 8:**
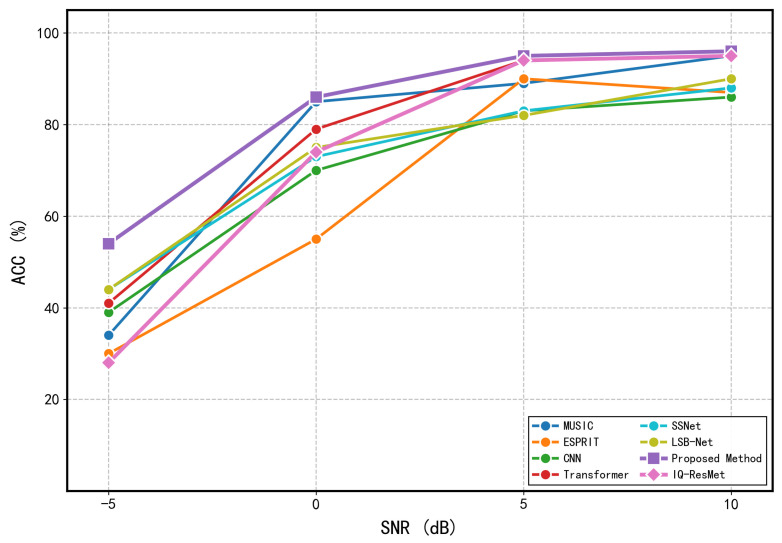
Accuracy of different models across SNR levels (two sources).

To establish a theoretical benchmark, we compute the Cramér-Rao Lower Bound (CRLB) for the two-source scenario following [24]. The CRLB provides a lower bound on the variance of any unbiased estimator, serving as a fundamental performance limit for DOA estimation. For the uniform linear array with N=10 sensors, L=1024 snapshots, and two uncorrelated far-field sources, the CRLB for the *i*-th DOA parameter is given by:

(51) CRLB(θi)=σn22LReDHPA⊥D⊙RsAHR−1ARs−1ii

where $\sigma_{n}^{2}$ is the noise variance, *L* is the number of snapshots, $A=[a\left(\theta_{1}\right),a\left(\theta_{2}\right)]$ is the N×2 array manifold matrix, $D=[\partial a\left(\theta_{1}\right)/\partial \theta_{1},\partial a\left(\theta_{2}\right)/\partial \theta_{2}]$ is the matrix of steering vector derivatives with respect to each DOA, $P_{A}^{⊥}=I-A{\left(A^{H}A\right)}^{-1}A^{H}$ is the orthogonal projector onto the noise subspace, $R_{s}$ is the source covariance matrix, and $R=AR_{s}A^{H}+\sigma_{n}^{2}I$ is the array covariance matrix. The RMSE of any unbiased estimator satisfies $\mathrm{RMSE}\left(\theta_{i}\right)\geq \sqrt{\mathrm{CRLB}(\theta_{i})}$. The CRLB curves, obtained by averaging over multiple random realizations of source angles. To comprehensively evaluate the performance of different methods in two-source scenarios, we conducted comparative experiments from both estimation accuracy and reliability perspectives. Figure 7 compares the RMSE of different methods across various SNR conditions, including MUSIC, ESPRIT, IQ-ResNet, the method by Zheng et al. [25], the proposed method, and the derived CRLB, highlighting the superior estimation accuracy of our approach. Unless otherwise specified, the minimum angular separation between the two sources is set to $20^{{}^{\circ}}$, and all subsequent data generation follows this configuration. In addition, we assess the reliability of angle estimation using a strict success criterion that requires both DOA estimates to have absolute errors below $0.5^{{}^{\circ}}$. The resulting accuracy curves are presented in Figure 8.

**Figure 9:**
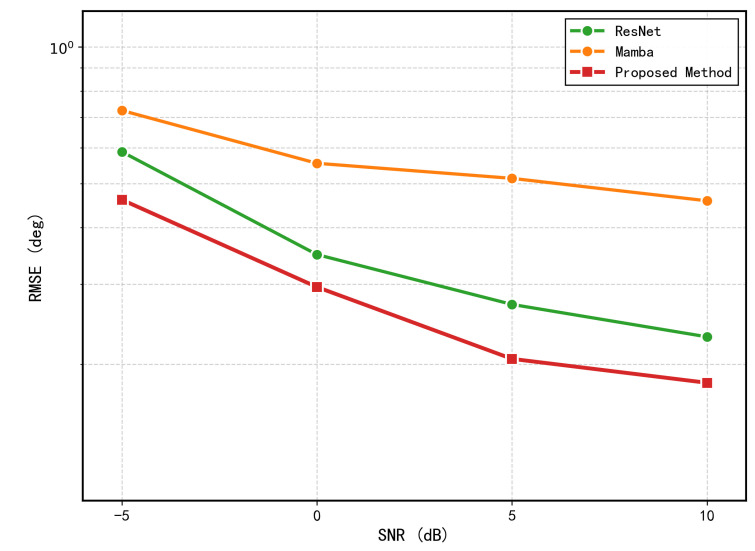
RMSE comparison of different ablation variants across SNR levels (two sources).

To evaluate the performance of different modules, we conducted an ablation experiment. Specifically, we compared the full model against two single-branch variants: (1) ResNet-only; and (2) Mamba-only. All variants were trained and tested under identical conditions. The result shows in Figure 9.

MUSIC is a spectral peak search method, and its angle estimation performance is limited by the predefined search grid and the requirement of prior knowledge of the number of sources. When two sources are closely spaced, the spectral peaks tend to merge, leading to significant estimation errors. In contrast, the proposed deep learning method also requires prior knowledge of the number of sources but learns to directly regress the DOA angles from the covariance matrix. To evaluate the resolution capability of both methods under challenging conditions, we conducted closely-spaced source estimation experiments. Specifically, we randomly generated 100 two-source scenarios for each of five angular separations: $1^{{}^{\circ}}$, $2^{{}^{\circ}}$, $3^{{}^{\circ}}$, $4^{{}^{\circ}}$, and $5^{{}^{\circ}}$, with both source angles uniformly sampled from $\left[-60^{{}^{\circ}},60^{{}^{\circ}}\right]$ while maintaining the specified separation between them. The SNR was uniformly distributed across the range of −5 dB to 10 dB, and 1024 snapshots were used for each sample. MUSIC was applied with a $0.05^{{}^{\circ}}$ search grid. Table 6 reports the RMSE averaged over the 100 scenarios for each angular separation, across all SNR conditions. The results demonstrate that the proposed method maintains a consistently low RMSE even at very small separations, whereas MUSIC suffers from severe performance degradation due to merging spectral peaks at closely spaced angles.

**Figure 10:**
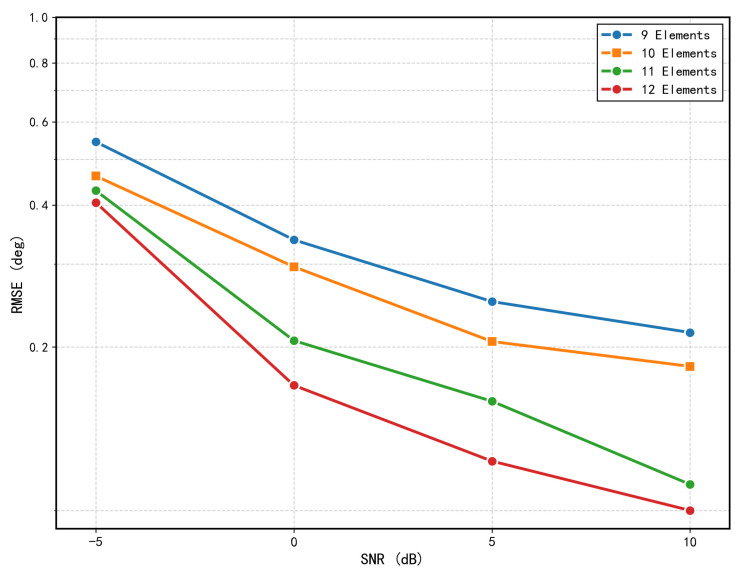
RMSE comparison of the proposed method across different numbers of array elements.

It should be noted that the current model is specifically trained on data generated from a 10-element uniform linear array (ULA), and therefore cannot be directly applied to ULAs with a different number of elements without retraining. This is because both the ResNet branch (which expects a fixed input size after interpolation) and the Mamba branch (which operates on a fixed sequence length of 10) are inherently tied to the number of array elements used during training. However, the proposed architecture is general and can be readily adapted to other element counts by simply adjusting the model structure and retraining on data generated from the corresponding array setup. To validate this flexibility, we trained separate instances of the proposed model for 9, 10, 11, and 12 array elements. As shown in Figure 10, the RMSE performance improves consistently as the number of array elements increases, which is expected due to the greater spatial diversity provided by larger arrays. This demonstrates that the proposed framework can be successfully extended to different element counts with minimal architectural modifications.

### 3.4. Results for Three-Source Scenario

**Figure 11:**
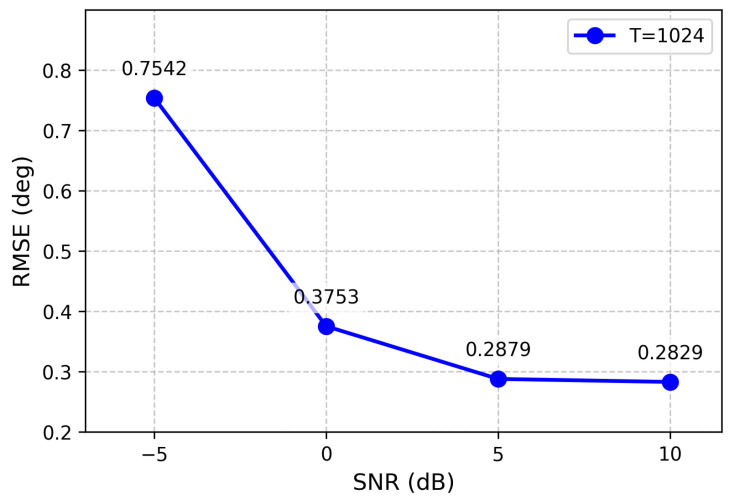
RMSE of the proposed method under different SNR levels (three sources).

Three-source RMSE results are shown in Figure 11.

**Figure 12:**
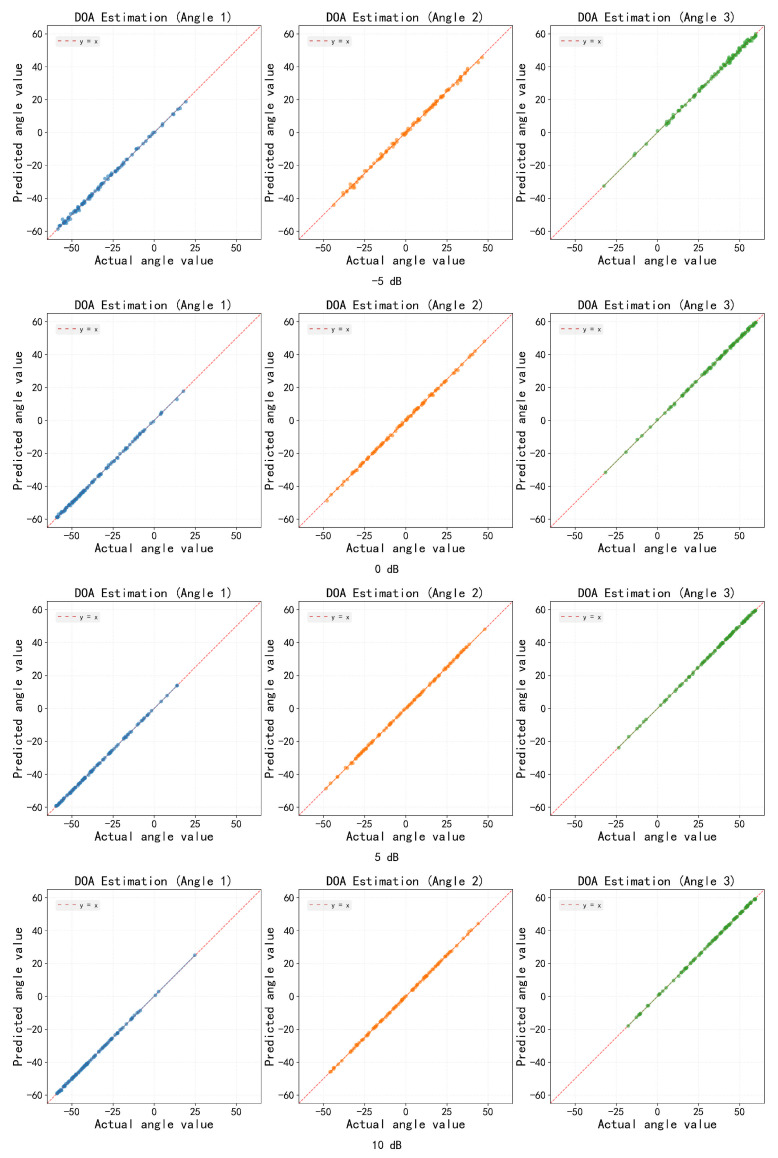
Scatter plots of the proposed method under different SNR levels (three sources).

The estimation characteristics of the proposed model in three-source scenarios are further evaluated through the scatter distribution of the estimated DOAs at four different SNR levels (−5, 0, 5, and 10 dB), as shown in Figure 12. These scatter plots provide an intuitive visualization of the estimation performance under varying noise conditions, clearly illustrating how the estimated angles converge toward the true values as SNR increases. Even at low SNR levels, the estimates form distinct clusters without significant bias, demonstrating the robustness and reliability of the proposed method.

**Figure 13:**
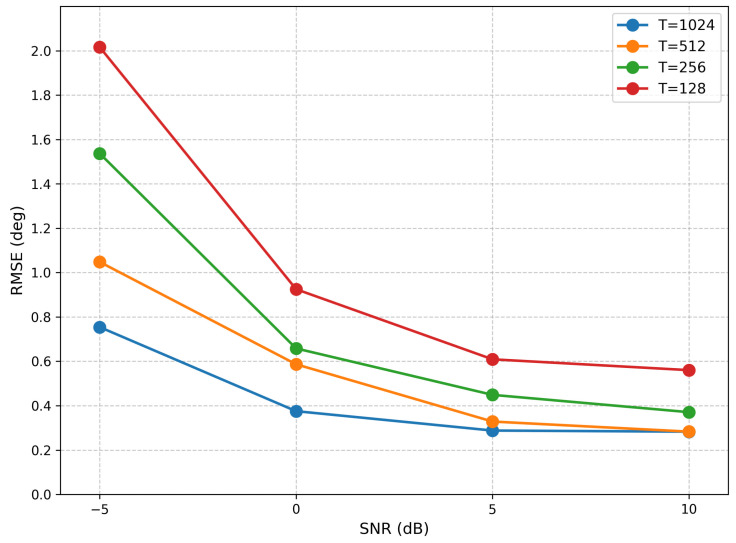
RMSE from different snapshots (three sources).

For the three-source scenario, the influence of snapshot number on estimation accuracy is illustrated in Figure 13, which presents the RMSE results across four snapshot settings (1024, 512, 256, and 128). The results demonstrate that the proposed method maintains robust performance even with limited snapshot numbers.

**Figure 14:**
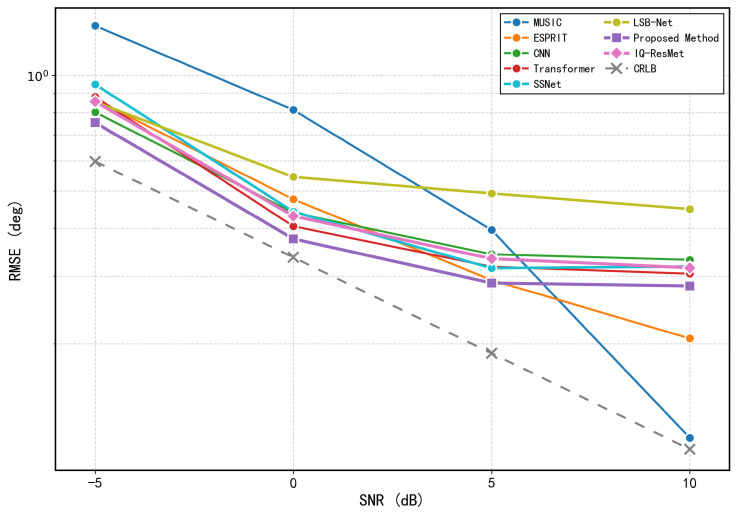
RMSE comparison of different methods under various SNR conditions (three sources).

**Figure 15:**
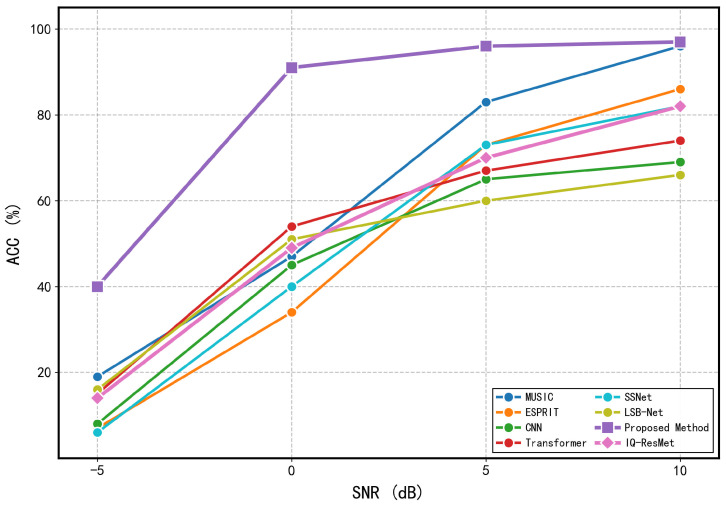
Accuracy of different models across SNR levels (three sources).

Figure 14 compares the RMSE of different methods in three-source scenarios, including MUSIC, ESPRIT, IQ-ResNet, and the proposed methods by Zheng et al. [25] and this work, highlighting the superior accuracy of our method. Figure 15 presents the success rates under a strict criterion requiring all three DOA estimates to have absolute errors below 0.5°, confirming that our method maintains consistently higher success rates across all SNR levels.

## 4. Discussion

The experimental results validate the effectiveness of the proposed ResNet-Mamba-MLP fusion framework for the DOA estimation task. The method demonstrates robust performance across a SNR range of −5 dB to 10 dB, maintaining a low Root Mean Square Error (RMSE) even under low SNR conditions. This showcases the model’s reliable estimation capability when signals are severely contaminated by noise. Furthermore, the RMSE analysis in single-source scenarios indicates that the proposed method achieves stable angle estimation under various SNR conditions.

Scatter plot analysis reveals the estimation characteristics of the model in multi-source scenarios. In the two-source case, under low SNR conditions, the estimated values are concentrated near the diagonal line without systematic bias, and they gradually converge as the SNR increases. This confirms the model’s ability to extract precise angular information from noisy observations. The scatter distribution in the three-source scenario further validates the generalization capability of the proposed method: even under a low SNR of −5 dB, the estimates for the three sources still form clear clusters, without the source loss or angular confusion commonly observed in traditional methods. As the SNR increases, the estimated points gradually converge towards the true values, and the scatter distribution becomes more compact. This indicates that the dual-branch architecture can effectively manage the mutual interference among multiple sources, maintaining good angular resolution even in complex signal environments.

Comparative analysis shows that the performance advantage of the proposed method is most pronounced in the most challenging scenarios. Under low-to-medium SNR conditions, the proposed method significantly outperforms both traditional methods like MUSIC and ESPRIT, as well as existing deep learning baselines, with a particularly notable reduction in estimation error. Only in the high-SNR region, such as when the SNR reaches 10 dB and the covariance estimate is sufficiently accurate, do the classical methods achieve performance comparable to the proposed method.

## 5. Conclusions

In this paper, a ResNet-Mamba hybrid deep learning framework for DOA estimation has been proposed. A dual-branch collaborative architecture was designed to jointly model spatial structure and sequential dependencies. The covariance matrix was processed as a 2D image by the ResNet branch to extract local spatial correlations, while sequential modeling along the array dimension was performed by the Mamba branch to capture long-range dependencies and phase progression patterns via its selective state-space mechanism. The complementary features were fused after layer normalization and passed to an MLP for DOA regression. To address the performance degradation of traditional methods in low SNR environments, robust estimation under noise-contaminated conditions was achieved by fusing local spatial features with global sequential dependencies. Extensive experiments on a 10-element uniform linear array dataset demonstrated that the proposed model significantly outperforms MUSIC, ESPRIT, and deep learning baselines in low SNR and limited snapshot scenarios, with substantial gains in estimation accuracy and success probability. Under higher SNR conditions, classical methods remained competitive, while comparable performance was maintained by the proposed framework. Evaluation based on strict success criteria further validated the method’s reliability across SNR levels, and sensitivity analysis confirmed robust performance under varying array configurations. The success of the proposed approach was attributed to the complementary nature of the dual-branch architecture, validated by ablation studies showing that the fusion of local spatial features and global sequential dependencies yields synergistic gains. Training stability and information preservation were ensured by the layer-normalized feature fusion mechanism, and favorable computational efficiency was demonstrated. Future work will focus on incorporating physical constraints, validating the model on real-world experimental data, and developing lightweight variants for edge deployment.

## Figures and Tables

**Table 1:** Comparison of existing deep learning-based DOA estimation methods.

Method	Advantages	Disadvantages
FCN-based [12]	Simple structure, low latency	Disrupts spatial proximity, quadratic parameter growth
CNN-based [14]	Preserves spatial structure	Limited receptive field, weak long-range dependency
RNN [19]	Captures temporal/sequential dependencies	Vanishing gradient, ignores spatial structure
Proposed method	Dual-branch, robust at low SNR, lightweight	Training slightly more complex

**Table 2:** Trainable parameter counts for each module.

Module	Number of Parameters
ResNet Branch	3,976,130
Mamba Branch	403,584
Fusion MLP	658,178
Total	5,037,892

**Table 3:** ResNet feature extractor configuration.

Item	Configuration
Input image size	2×224×224
Number of convolutional layers	5
Initial channels	48
Final channels	768
Kernel sizes	7, 5, 3, 3, 3
Pooling	Adaptive average pooling
Regression head	768→384→192→2
Dropout rate	0.2/0.1

**Table 4:** Mamba feature extractor configuration.

Item	Configuration
Input feature dimension	20
Sequence length	10
Hidden dimension	128
Number of Mamba blocks	4
Expand ratio	2
Gating mechanism	Input-dependent gating with sigmoid activation
Dropout rate	0.1
Activation function	GELU
Normalization	LayerNorm within each block
Pooling	Mean + Max concatenation
Output dimension	256

**Table 5:** Fusion MLP regressor configuration.

Item	Configuration
Input dimension	1024
Hidden layer 1	512
Hidden layer 2	256
Output dimension	2
Number of layers	3 fully-connected layers
Activation function	GELU
Normalization	LayerNorm after each hidden layer
Dropout rate	0.1

**Table 6:** RMSE comparison of the proposed method with MUSIC across different DOA angles.

DOA Angle (°)	Proposed Method	MUSIC
1	0.4003	26.4148
2	0.2056	19.4146
3	0.2594	14.2466
4	0.3199	5.8368
5	0.3271	0.3030

## Data Availability

The data supporting the findings of this study are available from the corresponding author upon reasonable request.

## Abbreviations

The following abbreviations are used in this manuscript:

DOA	Direction of Arrival
SNR	Signal-to-Noise Ratio
ACC	Accuracy
ResNet	Residual Network
MUSIC	Multiple Signal Classification
ESPRIT	Estimation of Signal Parameters via Rotational Invariance Techniques
